# Identification of High-Risk Pregnancies in a Remote Setting Using Ambulatory Blood Pressure: The MINDI Cohort

**DOI:** 10.3389/fpubh.2020.00086

**Published:** 2020-03-24

**Authors:** Doris González-Fernández, Emérita del Carmen Pons, Delfina Rueda, Odalis Teresa Sinisterra, Enrique Murillo, Marilyn E. Scott, Kristine G. Koski

**Affiliations:** ^1^School of Human Nutrition, Faculty of Agricultural and Environmental Sciences, McGill University (Macdonald Campus), Ste-Anne-de-Bellevue, QC, Canada; ^2^Department of Nutritional Health, Ministry of Health, Panama City, Panama; ^3^“Comarca Ngäbe-Buglé” Health Region, Ministry of Health, San Félix, Panama; ^4^“Panamá Norte” Health Region, Ministry of Health, Panama City, Panama; ^5^Department of Biochemistry, University of Panama, Panama City, Panama; ^6^Faculty of Agricultural and Environmental Sciences, Institute of Parasitology, McGill University, Ste-Anne-de-Bellevue, QC, Canada

**Keywords:** MAP, pulse pressure, symphysis-fundal height, micronutrients, protein status, uro-genital infections, nematodes

## Abstract

**Background:** Ambulatory blood pressure is a potential tool for early detection of complications during pregnancy, but its utility in impoverished settings has not been assessed. This cross-sectional study aimed to determine whether maternal infections, nutrient deficiencies and inflammation (MINDI) were associated with four measures of maternal blood pressure (BP) and to determine their association with symphysis-fundal-height (SFH).

**Methods:** Environmental and dietary factors, intake of iron and a multiple-nutrient supplement (MNS), markers of inflammation, protein, anemia, folate, vitamins B_12_, A and D status, and urogenital, skin, oral and intestinal nematode infections were measured in indigenous pregnant Panamanian women. Stepwise multiple linear and logistic regression models explored determinants of systolic and diastolic blood pressure (SBP, DBP), hypotension (SBP < 100 and DBP < 60), mean arterial pressure (MAP), elevated MAP (eMAP), and pulse pressure (PP). Associations of BP with intestinal nematodes and with SFH Z scores (≥16 wk) were also explored.

**Results:** Despite absence of high SBP or DBP, 11.2% of women had eMAP. Furthermore, 24.1% had hypotension. Linear regression showed that hookworm infection was associated with higher SBP (*P* = 0.049), DBP (*P* = 0.046), and MAP (*P* = 0.016), whereas Ascaris was associated with lower DBP (*P* = 0.018) and MAP (*P* = 0.028). *Trichomonas* was also associated with lower SBP (*P* < 0.0001) and MAP (*P* = 0.009). The presence of *Trichuris* (OR: 6.7, 95% CI 1.0–44.5) and folic acid deficiency (OR: 6.9, 95% CI 1.4–33.8) were associated with increased odds of eMAP. The odds of low BP was higher in the presence of *Ascaris* (OR: 3.63 ± 2.28, *P* = 0.040), but odds were lowered by MNS (OR: 0.35 ± 0.11, *P* = 0.001), more intake of animal-source foods/wk (OR: 0.7, 95% CI 0.5–0.9) and by higher concentrations of IL-17 (OR: 0.87 ± 0.05, *P* = 0.016).

**Conclusion:** MINDI were bi-directionally associated with blood pressure indicators. In this MINDI cohort, infections, nutrients and cytokines both raised, and lowered BP indices. The presence of eMAP identified pregnant women at risk of hypertension whereas low PP was associated with lower SFH. Therefore, MAP and PP may help in detecting women at risk of adverse pregnancy outcomes in settings with limited access to technology.

## Introduction

Appropriate pregnancy follow-up is a major challenge in settings where access to health care is limited. Fortunately, maternal blood pressure (BP) is easily measured even in remote settings. It is considered as an indicator of quality antenatal care (1) and useful in predicting hypertensive disorders of pregnancy (HDPs), preterm-birth and small-for-gestational-age (SGA) infants (2). In addition to systolic and diastolic blood pressure (SBP and DBP), both mean arterial pressure (MAP), and pulse pressure (PP) (3) also have clinical value. SBP, DBP (4), MAP (5), and PP (6, 7) are known to be elevated in early pregnancy before the development of HDPs whereas low DBP and MAP have been associated with poor fetal outcomes (8, 9). However, clinical interpretation may be misleading if co-existing conditions such as multiple infections, nutritional deficiencies, and inflammation (MINDI) modulate the relationship between blood pressure and pregnancy outcomes, including poor intrauterine growth.

Maternal nutrient deficiencies have been associated with both abnormal maternal blood pressure and decreased fetal growth. Maternal protein-energy malnutrition can lead to intra-uterine growth retardation (IUGR) (10), and low protein intake (<65 g/d) more than tripled the risk of HDPs (11). Although severe anemia is one of the main factors associated with HDPs (12), elevated hemoglobin (≥132 g/L) was positively associated with SBP and DBP (13), and hematocrit was positively correlated with MAP and with peripheral vascular resistance (14). With respect to vitamins, increase in vitamin D concentration by ≥ 30 nmol/L from the first to third trimester lowered the odds of preeclampsia (15). Homocysteine, which increases in response to low folate or vitamin B_12_ (16), was positively associated with MAP during pregnancy (17), and has been implicated in the physiopathology of HDPs and IUGR (16). Studies on vitamin A are contradictory. Women with retinol concentration >1.08 μmol/L had decreased risk of HDPs in Peru (18) whereas retinol concentrations > 1.05 μmol/L increased the risk of HDPs in Zimbabwe (19). Together, these studies highlight the potential associations of nutrients with blood pressure, but no studies have explored the combined impact of co-existing nutrient deficiencies on maternal/fetal health.

The limited evidence to date shows that common infections in impoverished rural populations affect blood pressure and pregnancy outcomes in a pathogen-specific manner. A prospective-cohort study showed that acute malaria lowered SBP, DBP and MAP but not PP (20) whereas both urinary infections (12) and the protozoan tissue parasite, *Toxoplasma gondii* (21) have been shown to increase odds of hypertension in pregnancy. The nature of the relationship between infection and blood pressure may depend on whether the infection induces a pro- or anti-inflammatory response. Inflammation modulates blood pressure in pregnancy (22) as evidenced by the association of HDPs with inflammatory markers including C-reactive protein (CRP) (23, 24) and with two pro-inflammatory cytokines, interleukin (IL)-6 and tumor necrosis factor (TNF)-α (25). In contrast, down-regulation of the pro-inflammatory response by IL-10 and T-regulatory cells (T-regs) has been shown to reduce the pathology of HDPs (26).

In areas where infections and nutrient deficiencies are common, a direct association between maternal blood pressure and fetal growth is difficult because of the absence of ultrasound, the current gold standard for fetal biometry assessment (27). However, SFH is widely used in Latin America for estimating fetal growth using PAHO standards (28). New standards for symphysis-fundal height (SFH) according to gestational age (GA) were recently developed by the INTERGROWTH project based on a large database of pregnant women from Brazil, China, India, Italy, Kenya, Oman, United Kingdom, and United States (29). The INTERGROWTH Project now allows SFH to be used as a first level screening tool for assessing the feto-maternal unit when ultrasound is not available. Currently there is no information on the application of new INTERGROWTH standards in Latin America.

In collaboration with the Panamanian Ministry of Health, we had previously collected a large dataset from our MINDI cohort of indigenous pregnant women in a region where HDPs and low birthweight are major public health concerns (30). Since BP measurements have been used in the early prediction of HDPs (31) and SFH has shown a sensitivity of 52% and a specificity of 92% in the detection of IUGR (28), and given that both BP and SFH, were the only clinical tools available to detect women at risk of adverse pregnancy outcomes, a comprehensive analysis of the database was used to identify associations among BP measurements, MINDI, and the recently released INTERGROWTH standards for symphysis-fundal height (29). The objectives of this cross-sectional study using our MINDI cohort were to determine if multiple infections, nutrient deficiencies and inflammation were associated with measures of maternal blood pressure [SBP, DBP, MAP, PP, elevated MAP (eMAP), and low blood pressure (<100/60 mmHg)] and to determine which BP measurements were associated with SFH using the new INTERGROWTH standards. We also investigated associations of parasitic infections, in particular intestinal nematodes with BP measurements, given previous bidirectional associations of intestinal nematodes with CRP during pregnancy in this same population (32).

## Methods

### Study Population

This cross-sectional study was conducted between August and December 2010 in the extremely impoverished Ngäbe-Buglé indigenous population in western Panama. In Panama, 13% of maternal mortality has been attributed to HDPs (30). Based on government statistics, 6.7% of institutional deliveries are low birthweight infants at the country level (33), whereas low birthweight rates are as high as 14% in the Ngäbe population (34). We have previously reported that pregnant women in our study had a high prevalence (>50%) of several vaginal and intestinal infections as well as oral, skin and urinary tract infections (35). Despite distribution of iron and micronutrient supplements by the Ministry of Health, >40% of these women had deficiencies of vitamins A, D and B_12_, and CRP was elevated in 16.4% of these women (32). In the study area ~40% of deliveries occurred at home, the zone was not endemic for malaria. Women were considered for the study if consulting for normal pregnancy follow up, and they did not present with signs of distress. The presence of HIV and gestational diabetes had been ruled-out by the Ministry of Health (35).

### Ethics and Recruitment

This study was carried out in accordance with the recommendations of the Operational Guides of Bioethics in Research, of the Gorgas Memorial Institute Research Bioethics Committee (Panama). Ethics approval was obtained from the Gorgas Memorial Institute and from McGill University. Participants gave written informed consent in accordance with the Declaration of Helsinki.

Indigenous women had government-driven incentives to attend pregnancy follow-up. The Panamanian Ministry of Health ran a program of food vouchers that were given to pregnant women when they attended pregnancy follow-up at the local health center. All pregnant women attending normal pregnancy follow-up in 14 health centers of the Ngäbe-Buglé region in Chiriquí, Panama were invited to participate. Most women lived within 2 h walking-distance from community health centers. For the study, nurses working at health centers informed the community of the visit of the research team during scheduled pregnancy follow-up days, and that they could get a laboratory evaluation without needing to travel to the regional hospital, located 1–3 h by car. Based on the annual estimate of 2,127 live-births in the Ngäbe-Buglé community in 2010 (33), we recruited >90% of the pregnant women in the region at the time of the study. Given the prevalence of 4.5% for HDPs in a population of women ≤ 20 y from Panama (36), and on successful use of ambulatory blood pressure to detect a prevalence of 4.7% HDPs (37), we estimated that a sample size of 67 pregnant women would allow us to detect women at risk of HDPs. Based on 6.7% prevalence of low birthweight (33), our proxy for impaired fetal growth, we estimated that 92 pregnant women would be sufficient to detect small SFH with a level of confidence of 95%. We were able to recruit 213 pregnant women, 174 of whom were beyond 16 wk of pregnancy, the minimal GA for SFH to be compared with the INTERGROWTH standards (29).

### Questionnaire and Physical Examination

Pregnant women answered questions about reproductive history, daily intake of iron supplements (60 mg tablets), multiple nutrient supplements (MNS) (tbsp/d), weekly intake of animal-source foods, fruits and vegetables, field work (hrs/d), and wood smoke exposure (h/d). Anthropometry was measured and the body mass index (BMI) was classified as underweight, normal or overweight using Pan American Health Organization standards for GA in pregnant women (28). When used as a continuous variable, maternal BMI was calculated as maternal weight/height^2^.

Maternal blood pressure (Omron HEM-790IT® automatic BP monitor) was measured in a sitting position, and re-measured if elevated (SBP ≥ 140 or DBP ≥ 90 mmHg), in which case the second measurement was recorded (38, 39). As most women arrived to health centers after considerable walking, at least 15 min rest were allowed before examination. Intake of coffee and wood smoke were recorded. URISCAN® dip-stick strips on a Miditron-M semi-automated reflectance photometer were used for semi-quantitative measurements of urinary specific gravity (USG) and protein. Hypertension was defined as a combination of elevated SBP and DBP. Mothers were considered to have HDPs if they presented with hypertension and dipstick-proteinuria ≥ 1+ or if symptoms of preeclampsia were present after week 20 of pregnancy (40). Low blood pressure was defined as SBP < 100 and DBP < 60 mmHg, corresponding to values <10th centile of SBP and DBP from a large cohort of pregnant women (*n* = 10,327) (2). MAP was calculated as DBP + 1/3 (SBP-DBP) (31). Trimester-specific cutoffs for elevated MAP (eMAP) in pregnancy were >87 mmHg (10–18 wks), >84 mmHg (18–34 wks), and >86 mmHg (after 34 wks) (41), and low MAP was defined as <70 mmHg (42). Pulse pressure was calculated as the difference between SDP and DBP (3). Cut-offs for elevated PP (>68 mmHg) and low PP (<42 mmHg) were based on a large population study of normal pregnant women (43).

SFH was measured after the mother had recently emptied her bladder and while she was in a supine position. A non-elastic tape was placed at the upper border of the symphysis pubic bone, and straightened over the uterus until reaching the fundus. The cubital edge of the hand was used to hold the tape at the point of the fundus while it was turned to see the numbers; the value was recorded to the nearest complete half centimeter. The INTERGROWTH standards for SFH were used to calculate SFH Z-scores and centiles in women with GA ≥ 16 weeks (*n* = 177) (29).

### Infections

Maternal infections were evaluated both clinically and using laboratory tests as previously described (35). Briefly, skin lesions compatible with scabies and oral caries were detected during the clinical exam (0 = no, 1 = yes), and bacteriuria based on microscopic analysis of centrifuged urine was scored 0 (absent), 1+ (few), 2+ (moderate) and 3+ (abundant). Vaginal Gram smears were assigned semi-quantitative scores (0–4) for *Lactobacillus, Bacteroides/Gardnerella* and *Mobiluncus*, and these scores were used to diagnose bacterial vaginosis (BV) based on a Nugent score ≥7, calculated as *Bacteroides/Gardnerella* score + (4 – *Lactobacillus* score) + (*Mobiluncus* score/2) (44). Semi-quantitative scores were similarly assigned for Gram-detected vaginal trichomoniasis and diplococcal infection, and for vaginal yeast detected by direct smears. Presence of intestinal nematodes (*Ascaris*, hookworm and *Trichuris*) was identified using direct microscopic fecal examination, Kato-Katz and Flotac® from the 120 women who provided stool samples, as previously described (35).

### Laboratory Analyses

#### Hematological Status and Inflammation

Complete red (RBC) and white blood cell (WBC) counts (BC-5500 Mindray Auto Hematology Analyzer) were performed. Anemia was defined as hemoglobin <110 g/L (45). Hematocrit was compared against normal ranges for the first (31–41%), second (30–39%), and third (28–40%) trimesters (46). To estimate the degree of hemoconcentration, hematocrit was classified in quantiles according to our population distribution: <25th quantile, 25–50th, 50–75th, and >75th quantiles (<33.2%, 33.2–35%, 35–36.9%, and >36.9%, respectively). Hypohydration was assessed as USG > 1,020 (47). Serum was analyzed for CRP using solid phase ELISA (MP Biomedicals, Orangeburg, NY) with a minimum detectable concentration of 0.95 nmol/L. Nine serum cytokines, IL-1β, IL-4, IL-6, IL-10, IL-12, IL-13, IL-17, interferon (IFN)-γ and TNF-α, were quantified by Luminex using a Human Cytokine/Chemokine Magnetic Bead Panel (Millipore Corporation Canada).

#### Maternal Nutritional Status

Serum samples were processed for folic acid and vitamin B_12_ concentrations using immunoelectro-chemiluminescence (MODULAR E170, Roche Diagnostics GmBH, Mannheim, Germany); for 25-OH vitamin D using the LIAISON, DiaSorin direct competitive chemiluminescence immunoassay; for vitamin A using HPLC (48); and for retinol binding protein (RBP) using Human RBP4 ELISA (MP Biomedicals) with a standard curve range between 0.14 and 100 ng/mL. Folic acid deficiency was defined as <10 nmol/L and vitamin B_12_ deficiency as <150 pmol/L (49). We used a cutoff for vitamin D deficiency of <50 nmol/L (50). Low vitamin A was defined as <1.05 μmol/L (51, 52) and low protein status as RBP <30 mg/L (53). Some women provided a sample of their coffee (*n* = 28), where caffeine was measured using HPLC (54).

### Statistical Analysis

All statistical analyses were performed using STATA 14 (StataCorp, TX, USA). Given that a few women did not provide urine (*n* = 5) or vaginal samples (*n* = 2), that the volume of serum samples was insufficient to process vitamin A analysis (*n* = 3) and cytokine assays (*n* = 1), and that only 120 women provided stool samples for intestinal nematode screening, multiple linear regression models included the STATA complete-case analysis function (55) which allowed us to both maximize the sample size of each final model and confirm the randomness of missing data using Little's chi-squared test (56).

Initial exploratory models for SBP, DBP, MAP, and PP were developed using stepwise multiple linear regressions accepting variables with *P* < 0.15 for six distinct clusters of variables: (1) environmental/supplementation variables (intake of animal-source foods, fruits and vegetables, coffee, intake of iron and MNS, field work, wood smoke); (2) RBC indices including hematocrit quantile and anemia; (3) inflammation indicators [WBC count and differential, neutrophil-lymphocyte ratio (NLR), CRP and cytokines]; (4) nutritional deficiencies (low protein and deficiencies of folic acid, vitamins B_12_, A and D); (5) infections with prevalence ≥10% (caries, scabies, and BV) as well as semi-quantitative scores for urinary bacteria, and vaginal *Bacteroides/Gardnerella, Mobiluncus, Lactobacillus*, trichomoniasis, diplococci and yeast; and (6) intestinal nematode infections (*Ascaris*, hookworm and *Trichuris*). We controlled for gestational age in all models. Maternal characteristics (age, parity, BMI category and urinary gravity ≤ 1,020) were also included if they had *P* < 0.15 in screening models.

Spearman correlations were calculated among independent variables within each set to avoid inclusion of significantly correlated variables in the same regression model. The independent occurrence of physiologically-related variables (low RBP with vitamin A deficiency and elevated CRP; anemia with low hematocrit) was tested using Chi^2^ analysis. Then, final stepwise multiple linear regression models were developed. Depending on the sample size for each final model, inclusion of 6–10 independent variables allowed us to have power of 0.80 and a medium effect size (57). If the stepwise process yielded more variables than permitted in the regression model given the sample size, the filter was lowered from 0.15 to *P* < 0.10 or *P* < 0.05 until the number of variables was appropriate for the sample size.

Linear regression models for SBP, DBP, MAP, and PP were run with (*n* = 120) and without (*n* = 213) intestinal nematodes. Given that MAP (but not other BP measurements) differed by trimester, multiple linear regressions were also run for MAP by trimester, with and without intestinal nematodes. We also confirmed the absence of collinearity based on a variance inflation factor (VIF) <10 and the stability of regression coefficients by a condition number <30. Significance of variables in the final models was set at *P* < 0.05. Only the final models are presented.

Variables associated with eMAP and low blood pressure (SBP <100 and DBP <60 mmHg) were explored using multiple logistic regression analysis, following the same sequence of steps as used for the multiple linear regressions.

For SFH Z-scores, Student's *T*-tests were used to establish differences between the presence/absence of abnormal blood pressures: eMAP (classified by GA), low MAP (<70 mmHg), low BP (<100/60 mmHg), and low PP (<42 mmHg). For assessing if SFH Z-score was associated with BP indicators, independent factor linear regression analyses were conducted. For each blood pressure indicator, the data were separated into three “factors” (factor 1: <10th centile; factor 2: 10–90th centile; factor 3: >90th centile) as determined by 10 and 90th centile values from our data (SBP, 90 and 117.6 mmHg; DBP, 51.4 and 72 mmHg; MAP, 65.6 and 86.6 mmHg; and PP, 30 and 51.6 mmHg). Regression models for SFH-Z-score were constructed using factor 2 as the reference factor. Reported coefficients compared women in factor 1 and in factor 3 with those in factor 2. Significance was set at *P* < 0.05. Associations were further tested after adjusting for two known risks for IUGR, low BMI and wood smoke exposure.

## Results

### Population Characteristics

Descriptive data are reported in Table 1. The population had a series of risk factors for adverse pregnancy outcomes, including pregnancy in adolescents and those over 35 y, and primiparous and grand-multiparous (≥5) pregnancies. Most women used wood as fuel for cooking and 82% had access to <7 portions/wk of animal source foods. Coffee intake was common, but the caffeine content of coffee was low, with a median (min-max) of 4.5 (0.3–24.5) mg/100 mL. The majority of women (67.1%) had a normal weight-for-height-for-gestational age, but low weight for GA (9.9%) and overweight (23%) were also present. Over 50% of women had vitamin B_12_ or D deficiency, and over 20% had low RBP, low vitamin A or folic acid deficiency. RBP concentration was not correlated with concentration of vitamin A (*r*_s_ = 0.04, *P* = 0.55, *n* = 212) or CRP (*r*_s_ = −0.10, *P* = 0.12, *n* = 212), but was correlated with B_12_ (*r*_s_ = 0.15, *P* < 0.03, *n* = 207) which is predominantly found in animal source foods. Of the two supplementation programs in the communities, iron tablets reached over 75% of women, and half the women took MNS, although at a median intake well below the recommended 6 tbsp/d. Despite use of supplements, over one-third of the women were anemic. Infections were extremely common and 97% of women had at least two infections. The most prevalent were vaginal infections, followed by intestinal nematodes, urinary, oral and skin infections (Table 1).

**Table 1:** Population characteristics in pregnant Ngäbe-Buglé women from rural Panama^a^.

**Maternal characteristics, *n* (%)**		**Nutritional characteristics**	
Age		Weight for height category, *n* (%)	
≤ 19 yrs	62 (29.1%)	Underweight	21 (9.8%)
≥35 yrs	28 (13.1%)	Overweight	49 (23.0%)
Trimester		**Serum nutrients**	
First	26 (12.2%)	Retinol-binding protein	
Second	80 (37.6%)	[median (min-max)]	4.8 (0.3–41.2)
Third	107 (50.2%)	<30 mg/L, *n* (%)	57 (26.9%)
Parity		Vitamin B_12_	
First gestation	60 (28.2%)	[median (min–max)]	100.0 (53.0–376.0)
≥5 gestations	69 (32.4%)	<150 pmol/L, *n* (%)	181 (84.9%)
Environment hazards		Vitamin D	
Wood smoke	195 (91.5%)	(mean ± SD)	44.5 ± 15.1
Fieldwork	110 (51.6%)	<50 nmol/L, *n* (%)	138 (64.8%)
**Blood pressure, (mean** **±** **SD)**		Vitamin A	
Systolic blood pressure, mmHg	102.8 ± 10.3	[median (min-max)]	1.2 (0.4–2.9)
First trimester	104.3 ± 10.3	<1.05 μmoll/L, *n* (%)	87 (41.4)
Second trimester	100.8 ± 10.9	Folic acid	
Third trimester	103.9 ± 9.7	[median (min-max)]	14.1 (6.3–45.4)
Diastolic blood pressure, mmHg	61.6 ± 8.5	<10 nmol/L, *n* (%)	51 (23.9%)
First trimester	63.3 ± 7.5	**Diet and supplementation**, ***n*** **(%)**	
Second trimester	59.8 ± 8.7	Animal source foods	
Third trimester	62.5 ± 8.5	<7 portions/wk	176 (82.6%)
Mean Arterial Pressure, mmHg		fruits-vegetables	
First trimester	77.6 ± 7.2	<7 portions/wk	179 (84.0%)
Second trimester	73.2 ± 8.5	Coffee intake	185 (86.8%)
Third trimester	76.3 ± 7.9	**Supplementation**	
Pulse pressure, mmHg		Iron, *n* (%)	163 (76.5%)
First trimester	40.7 ± 9.8	MNS, *n* (%)^2^	108 (50.7%)
Second trimester	41.0 ± 8.3	**Red blood cells**	
Third trimester	41.3 ± 8.5	Hemoglobin, g/L (mean ± SD)	111.6 ± 11.3
**Infections**, ***n*** **(%)**		Anemia, *n* (%)	81 (38.0%)
Caries	42 (19.7%)	Hematocrit (%) (mean ± SD)	34.8 ± 3.2
Scabies	37 (17.3%)	Mean corpuscular volume, fL (mean ± SD)	93.7 ± 6.0
Vaginal infections			
*Trichomonas vaginalis*	159 (75.3%)	**Urine analyses**, ***n*** **(%)**	
Bacterial vaginosis	128 (60.6%)	Urinary specific gravity > 1,020,	56 (26.9%)
*Lactobacillus*	113 (53.5%)	Bacteriuria ≥ 2+	54 (25.9%)
*Bacteroides/Gardnerella*	198 (93.8%)	**Intestinal parasites**, ***n*** **(%)**^**b**^	
*Mobiluncus*	174 (82.4%)	Hookworm	52 (56.6%)
Yeast	53 (24.9%)	*Ascaris*	39 (32.5%)
*Diplococcus spp*.	43 (20.3%)	*Trichuris*	15 (12.5%)

a *n = 208–213 for all, except for intestinal parasites (n = 120)*.

b *MNS: Multiple nutrient supplement containing in every 100 g, energy (400 kcal), protein (12.0 g), lipids (12–14 g), vitamin A (250 μg), vitamin E (10 mg), vitamin B_1_ (0.50 mg), vitamin B_2_ (0.50 mg), vitamin B_3_ (6.0 mg), vitamin B_6_ (0.50 mg), vitamin B_12_ (0.90 μg), folic acid (85 μg), iron (4.0 mg iron bisglycinate), zinc (4.5 mg amino chelated), calcium (100 mg), phosphorus (55 mg) and copper (400 μg) (58)*.

*Means ± SD or frequencies (%) are presented^a^*.

In our population, 27% of the women had USG>1,020 indicating hypohydration and 27% had RBP <30 mg/L indicating low protein status and suggesting low oncotic pressure. Moreover, despite the high prevalence of anemia (38%), all women had normal RBC count and most had normal (93.4%) or high (2.3%) hematocrit, further supporting the presence of hypovolemia in our population and suggesting that the normal hemo-dilution of pregnancy is not occurring.

**Figure 1:**
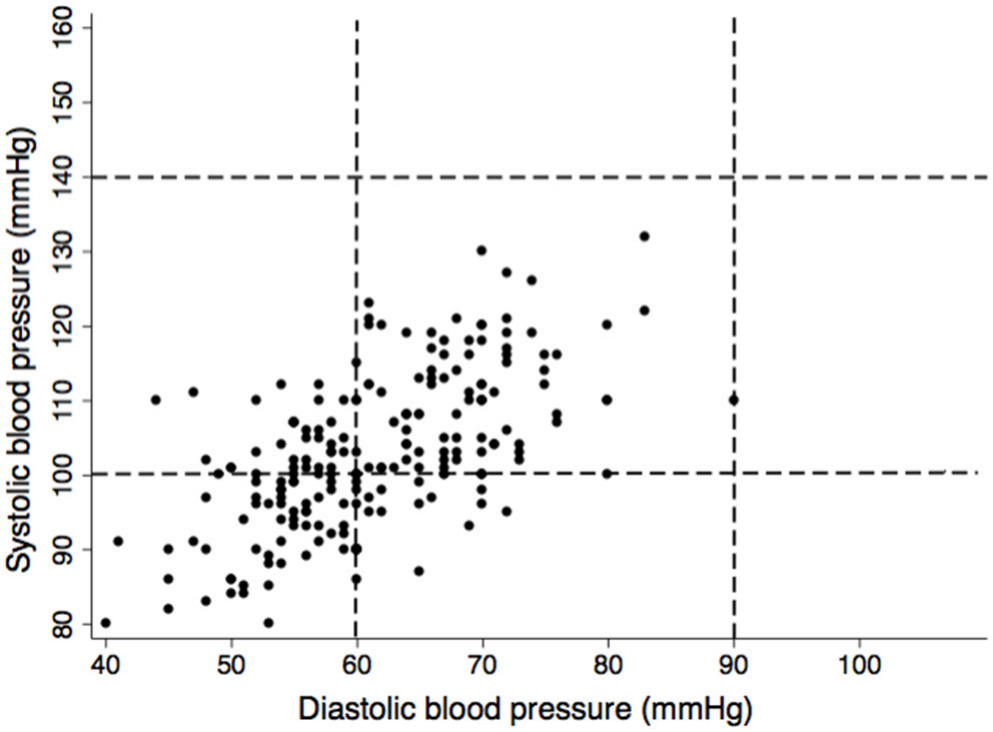
Scatter plot of systolic vs. diastolic blood pressure in 213 pregnant Ngäbe-Buglé women from Panama. Dashed lines represent blood pressure limits for hypertension during pregnancy (≥140 mmHg for SBP and ≥90 mmHg for DBP) (31), lower limits were defined following the most conservative cut-offs (<100 mmHg for SBP and <60 mmHg) for pregnant women (2).

Trimester-specific blood pressure measurements are described in Table 1. None of the pregnant women had high SBP or DBP (Figure 1). SBP, DBP, and PP did not differ by trimester, but MAP did (*P* = 0.035).

**Figure 2:**
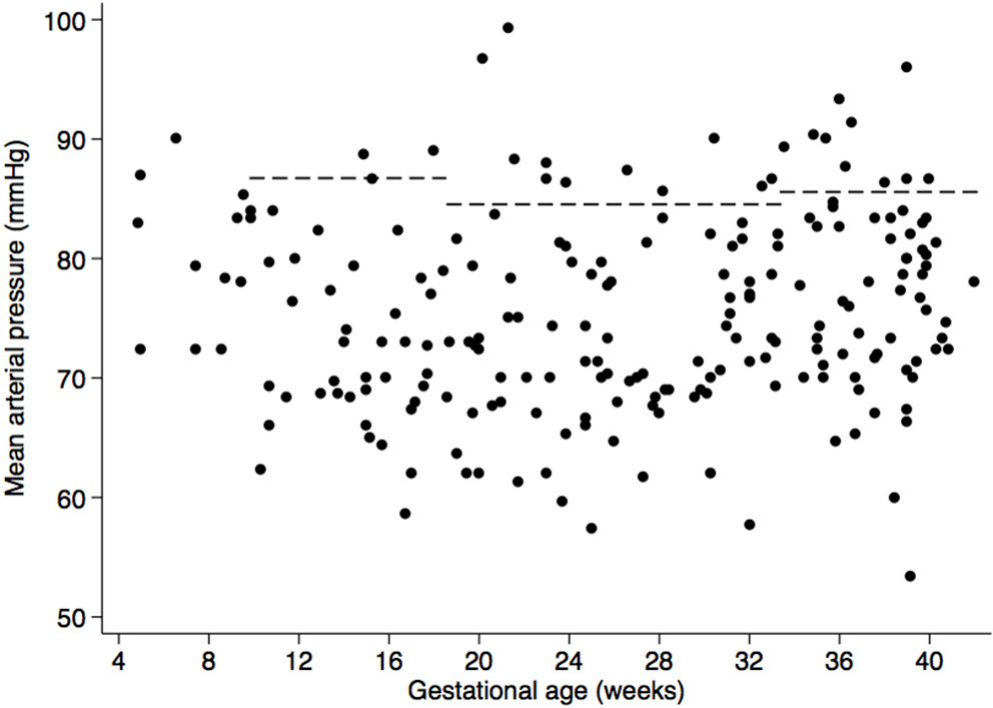
Scatter plot of mean arterial pressure (MAP) according to gestational age. Dashed lines represent cutoffs for elevated MAP: >87 mmHg between weeks 10–18, >84 mmHg in weeks 18–34, and >86 mmHg after week 34 (41).

**Figure 3:**
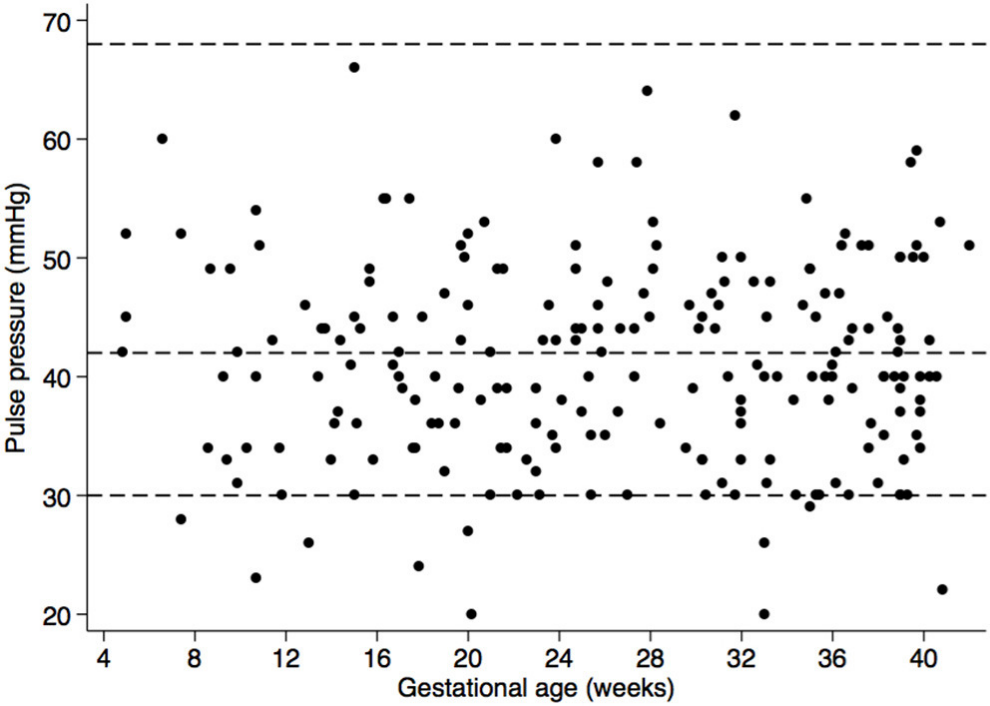
Scatter plot of pulse pressure (PP) according to gestational age. Dashed lines represent the cut-off for high PP (>68 mm Hg) and low PP (<42 mm Hg) based on findings from Ayala and Herminda (43), as well as the 10th centile in our data set (PP < 30 mm Hg).

Furthermore, none of the women presented with clinical manifestations of HDPs. Although urinary protein was found (2.9%), those women had urinary tract infection. However, using eMAP, risk of HDPs was detected in 11.3% women (Figure 2). With regard to PP, none of the women had elevated PP but 52.6% had low PP (Figure 3) and the 10th centile corresponded to a PP of 30 mmHg.

**Figure 4:**
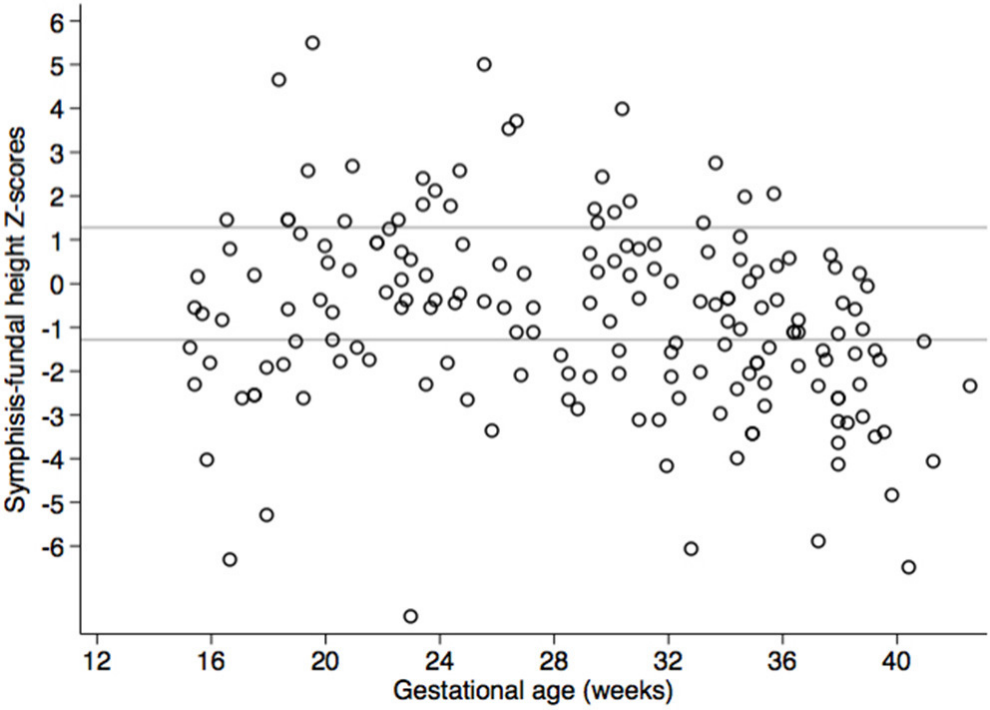
Scatter plot of symphysis-fundal height Z-scores based on INTERGROWTH standards for gestational age in pregnant Ngäbe-Buglé women from rural Panama. Reference lines mark the 10th centile, below which fetuses are considered to be small for gestational age, and the 90th centile, above which fetuses are considered large for gestational age (29).

Based on the INTERGROWTH standards for SFH, 50.6% (*n* = 88) of women had SFH measurements below the 10th centile and 9.2% (*n* = 16) had measurements above the 90th centile (Figure 4).

### Multiple Regression Models for Blood Pressure Measurements

Models including intestinal nematodes are shown in Tables 2, 3. Models not including intestinal nematodes with a larger sample size are provided as Tables S1, S2.

**Table 2:** Multiple stepwise linear regression models for systolic blood pressure (SBP) and diastolic blood pressure (DBP) in pregnant Ngäbe-Buglé women from rural Panama.

**A. SBP^**a**^^**,**^^**c**^**	**Coefficient ± SE**	***P***	**95% CI**	**β**	**Overall model**
GA, wks	0.09 ± 0.08	0.279	−0.07, 0.26	0.091	*P* < 0.0001 Adj. *R*^2^ = 0.313
Weight for height classification	4.08 ± 1.44	0.005	1.23, 6.93	0.239	
MNS, tbsp/d	1.53 ± 0.50	0.003	0.54, 2.52	0.262	
TNF-α, pg/mL	0.35 ± 0.09	<0.0001	0.16, 0.54	0.294	
Caries, presence	−3.3 ± 1.89	0.075	−7.14, 0.35	−0.142	
Scabies, presence	−4.28 ± 1.89	0.026	−8.03, −0.53	−0.182	
*Trichomonas*, presence	−6.99 ± 1.82	<0.0001	−10.62, −3.38	−0.309	
Hookworm, presence	3.21 ± 1.61	0.049	0.02, 6.40	0.162	
Constant	91.20 ± 4.18	<0.0001	82.91, 99.49		
**B. DBP** ^**b**^^**,**^ ^**c**^	**Coefficient** **±** **SE**	***P***	**95% CI**	**β**	**Overall model**
GA, wks	0.12 ± 0.09	0.173	−0.05, 0.29	0.139	*P* < 0.0001 Adj. *R*^2^ = 0.219
Weight for height classification	2.45 ± 1.30	0.063	−0.13, 5.04	0.166	
Urinary gravity >1,020	3.34 ± 1.72	0.054	−0.06, 6.75	0.164	
MNS, tbsp/d	0.98 ± 0.46	0.036	0.06, 1.91	0.195	
Iron supplement	−3.98 ± 2.08	0.059	−8.11, 0.14	−0.183	
RBP < 30 mg/L	4.44 ± 1.79	0.015	1.88, 8.00	0.214	
Folic acid < 10 nmol/L	2.85 ± 1.78	0.113	−0.69, 6.38	0.136	
*Ascaris*, presence	−3.77 ± 1.57	0.018	−6.88, −0.65	−0.207	
Hookworm, presence	3.04 ± 1.50	0.046	0.06, 6.02	0.177	
Constant	51.51 ± 3.58	<0.0001	44.40, 58.62		

a *n = 116. Missing data for TNF-α (1), Trichomonas (2), hookworm (93). Little's Chi-squared test for randomness of missing data, P = 0.698*.

*VIF = 1.11. Condition number = 15.29*.

*Variables that were included but had a P > 0.10: wood smoke exposure (0 = no, 1 = yes), hematocrit quantile, IL-13 (pg/mL), IL-17 (pg/mL), bacteriuria score, Ascaris (0 = no, 1 = yes)*.

b *n= 116. Missing data for RPB (1), urinary gravity (5), hookworm (93), Ascaris (93). Little's Chi-squared test for randomness of missing data, P = 0.197*.

*VIF = 1.18. Condition number = 15.23*.

*Variables that were included but had a P>0.10: hematocrit quantile, hemoglobin (g/L), IL-13 (pg/mL), vitamin D < 50 nmol/L, Trichomonas (0 = no, 1 = yes)*.

c *Unstandardized coefficient and standardized β. Binary variables (urinary gravity > 1,020, taking iron supplements and presence of infections) were included as 0 = no, 1 = yes*.

*GA, gestational age; MNS, multiple micronutrient supplement; RBP, retinol-binding protein*.

**Table 3:** Multiple stepwise linear regression models for mean arterial pressure (MAP), and pulse pressure (PP) in pregnant Ngäbe-Buglé women from rural Panama.

**A. MAP^a^^,^^c^**	**Coefficient ± SE**	***P***	**95% CI**	**β**	**Overall model**
GA, wks	0.07 ± 0.07	0.293	−0.06, 0.21	0.092	*P* < 0.0001 Adj. *R*^2^ = 0.294
Weight for height classification	3.24 ± 1.17	0.007	0.91, 5.56	0.233	
Urinary gravity > 1,020	3.03 ± 1.55	0.053	−0.03, 6.10	0.158	
MNS, tbsp/d	1.00 ± 0.42	0.020	0.16, 1.84	0.210	
TNF-α, pg/mL	0.17 ± 0.08	0.033	0.01, 0.33	0.178	
*Trichomonas*, presence	−4.02 ± 1.52	0.009	−7.03, −1.00	−0.217	
RBP < 30 mg/L	4.00 ± 1.61	0.015	0.81, 7.19	0.205	
*Ascaris*, presence	−3.13 ± 1.40	0.028	−5.91,−0.35	−0.182	
Hookworm, presence	3.28 ± 1.34	0.016	0.63, 5.94	0.204	
Constant	63.6 ± 3.4	<0.0001	56.9, 70.3		
**B. PP^b^^,^^c^**	**Coefficient** **±** **SE**	***P***	**95% CI**	**β**	**Overall model**
GA, wks	−0.05 ± 0.06	0.355	−0.18, 0.06	−0.066	*P* = 0.0007 Adj. *R*^2^ = 0.083
Age, yrs	−0.20 ± 0.08	0.013	−0.36, −0.04	−0.169	
MNS, tbsp/d	0.79 ± 0.38	0.040	0.03, 1.55	0.145	
Basophils, number/mm^3^	0.06 ± 0.04	0.110	−0.01, 0.14	0.109	
IL-17, pg/mL	−0.24 ± 0.07	0.002	−0.39, −0.09	−0.290	
TNF-α, pg/mL	0.27 ± 0.09	0.005	0.08, 0.46	0.261	
Constant	44.58 ± 2.83	<0.0001	39.0, 50.16		

a *n= 116. Missing data for TNF-α (1), RBP (1), Trichomonas (2), urinary gravity (5), hookworm (93), Ascaris (93). Little's Chi-squared test for randomness of missing data, P = 0.163*.

*VIF = 1.14. Condition number = 15.63. Variables that were included but had a P > 0.10: folic acid <10 nmol/L, hematocrit quantile, urinary protein score, hemoglobin (g/L), IL-13 (pg/mL), vitamin D < 50 nmol/L, vaginal yeast score*.

b *n = 206. Missing data for TNF-α (1), IL-17 (1), Mobiluncus and Bacteroides/Gardnerella (2), urinary gravity (5). Little's Chi-squared test for randomness of missing data, P = 0.928*.

*VIF = 1.36. Condition number = 13.31*.

*Variables that were included but had a P > 0.10: neutrophils (#), urinary gravity, Mobiluncus and Bacteroides/Gardnerella scores*.

c *MAP = DBP + 0.33 [SBP-DBP]; PP = SBP-DBP (3). Unstandardized coefficient and standardized β. Binary variables (urinary gravity > 1,020 and presence of infections) were included as 0 = no, 1 = yes*.

*GA, gestational age; BMI, body mass index; MNS, multiple micronutrient supplement; RBP, retinol binding protein*.

Several infections emerged in our multiple regression models for SBP (Table 2A), DBP (Table 2B) and MAP (Table 3A), and depending on the infection, were associated with either higher or lower BP measurements. First, presence of scabies was associated with lower SBP, and presence of trichomoniasis with lower SBP and lower MAP. Second, it is important to highlight that, whereas hookworm or *Trichuris* were consistently associated with higher blood pressure measures [SBP, DBP, MAP, eMAP models (Tables 2A,B, 3A, 4A)], *Ascaris* was associated with lower blood pressure measures, DPB, MAP, and low blood pressure (Tables 2B, 3A, 4B). Furthermore, despite the much lower sample size, inclusion of intestinal nematode variables increased the adjusted *R*^2^ of the final SBP, DBP and MAP models by more than 75% compared with models where intestinal nematode variables were not included (Table S1).

**Table 4:** Multiple logistic regression model for elevated mean arterial pressure (eMAP) and low blood pressure in pregnant Ngäbe-Buglé women from rural Panama.

**A. eMAP^a^^,^^c^**	**Odds Ratio ± SE**	***P***	**95% CI**	**Overall model**
Age, yrs	1.18 ± 0.07	0.003	1.06, 1.32	*P* = 0.0003 Pseudo *R*^2^ = 0.272
Folic acid < 10 nmol/L	6.98 ± 5.62	0.016	1.44, 33.79	
*Trichuris*, presence^c^	6.72 ± 6.48	0.048	1.01, 44.56	
Constant	0.0003 ± 0.0005	<0.0001	5.6^−6^, 0.01	
**B. Low blood pressure^b^^,^^c^**	**Odds Ratio** **±** **SE**	***P***	**95% CI**	**Overall model**
GA, wks	0.93 ± 0.03	0.036	0.87, 0.99	*P* < 0.0001 Pseudo *R*^2^ = 0.418
BMI, kg/m^2^	0.75 ± 0.07	0.002	0.62, 0.90	
Field work, h/d	0.60 ± 0.09	0.001	0.45, 0.81	
*Ascaris*, presence ^c^	3.63 ± 2.28	0.040	1.06, 12.41	
MNS, tbsp/d	0.35 ± 0.11	0.001	0.19, 0.65	
IL-17, pg/mL^3^	0.87 ± 0.05	0.016	0.78, 0.97	
IFN-γ, pg/mL^3^	1.08 ± 0.05	0.082	0.99, 1.18	
Hookworm, presence^*c*^	0.33 ± 0.21	0.080	0.09, 1.14	
Animal-source foods/wk	0.70 ± 0.10	0.012	0.54, 0.92	
Constant	62975.3 ± 190587	<0.0001	167.1, 2.47	

a *eMAP defined as >87 mmHg between weeks 10–18, >84 mmHg in weeks 18–34, and >86 mmHg after week 34 (41)*.

*n = 117. Missing data for IFNγ (1), IL-1β (1), IL-17 (1), RBP (1), bacteriuria (5), Ascaris (93), Trichuris (93), hookworm (93). Little's Chi-squared test for randomness of missing data, P = 0.486*.

*VIF = 1.03. Condition number = 7.76*.

*Variables that were included but had a P > 0.10: BMI category, NLR, MCV (fL), IL-1β (pg/mL), IL-17 (pg/mL), iron supplementation (mo), MNS supplementation (tbsp/d), RBP <30 mg/L, Ascaris (0 = no, 1 = yes), vaginal yeast (0 = no, 1 = yes), bacteriuria (0 = no, 1 = yes)*.

b *Low blood pressure defined as SBP < 100 and DBP < 60 mmHg (3)*.

*n = 119. Missing data for IFNγ (1), IL-17 (1), RBP (1), bacteriuria (5), Ascaris (93), Trichuris (93), hookworm (93). Two outliers of IL-17 were removed. Little's Chi-squared test for randomness of missing data, P = 0.579*.

*VIF = 1.32. Condition number = 27.44*.

*Variables that were included but had a P > 0.10: hematocrit quantile, green leafy vegetable intake/wk, iron supplementation (mo), folic acid <10 nmol/L, vitamin D (nmol/L), scabies (0 = no, 1 = yes)*.

c *Binary variables (folic acid <10 nmol/L and presence of infections) were included as 0 = no, 1 = yes*.

*GA, gestational age; BMI, body mass index; MNS, multiple micronutrient supplement; RBP, retinol binding protein; NLR, neutrophil-lymphocyte ratio; MCV, mean corpuscular volume*.

Regarding diet and nutrition, higher reported intake of MNS was associated with higher SBP (Table 2A), DBP (Table 2B), MAP (Table 3A), and PP (Table 3B), but was not associated with eMAP (Table 4A), whereas higher intake of animal-source foods was associated with decreased odds of BP < 100/60 (Table 4B). Also, two nutrient biomarkers were associated with BP: folic acid deficiency was associated with higher DBP and with increased odds of eMAP, and low RBP with higher DBP and MAP. Of note, folic acid deficiency was associated with higher MAP (Table S1), but lost its significance when intestinal nematodes were included (Table 3A).

Among inflammation indicators, two cytokines stood out in our models. TNF-α was associated with higher SBP (Table 2A), MAP and PP (Tables 3A,B); however, higher IL-17 was associated with lower PP, but only in the model without intestinal nematodes (Table 3B). Interestingly, IL-17 was not associated with SBP or DPB, but was associated with a lower likelihood of maternal BP < 100/60 (Table 4B).

Although we controlled all our models for GA (wks), given that MAP differed by trimester, we ran predictive models by trimester for MAP (Table S3). More cups/d of coffee were associated with lower MAP (*P* = 0.040) only in the first trimester, whereas higher Hb (*P* = 0.003) (model not shown), hematocrit (*P* = 0.014) (model not shown) as well as the red blood cell count (*P* < 0.0001) (Table S3) were associated with higher MAP also in the first trimester. In concordance, a higher hematocrit (*P* = 0.003) as well as hypohydration indicated by urinary gravity ≥ 1,020 (*P* = 0.009) were associated with higher MAP in the second trimester. In both the second (*P* = 0.009) and the third trimester (*P* = 0.003), MNS was associated with higher MAP and interestingly, *Ascaris* infection (*P* = 0.006) was associated with lower MAP but only in the third trimester (Table S3).

### Blood Pressure and SFH Z-Scores

**Figure 5:**
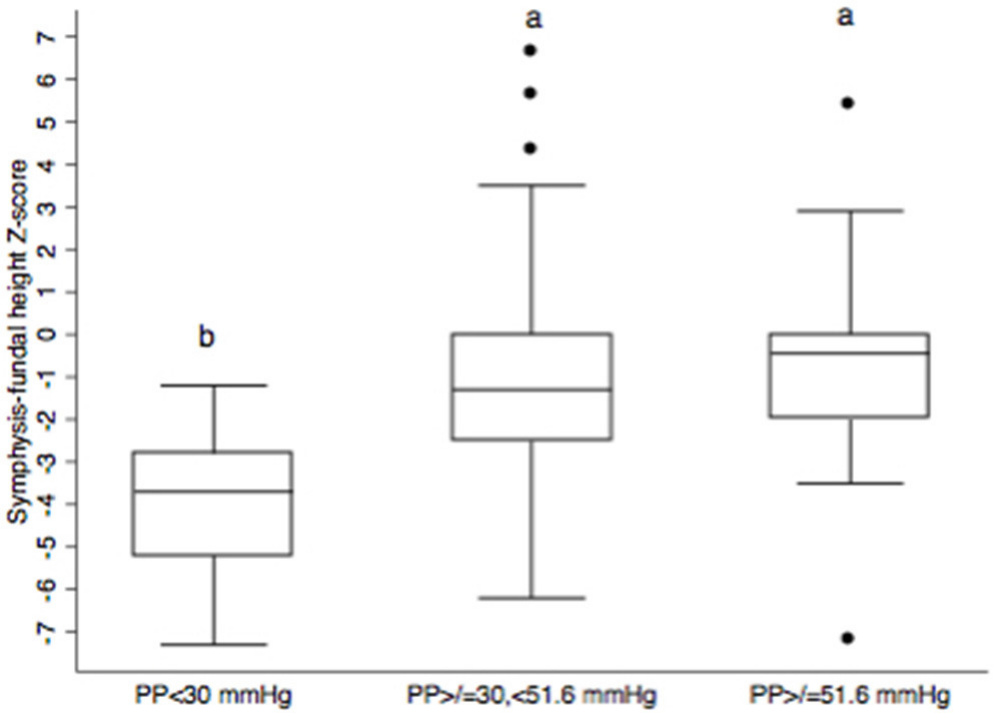
Box and whisker plots of symphysis-fundal height for three pulse pressure (PP) categories (<10th centile, 10–90th centile, and ≥90th centile) based on data from 177 pregnant Ngäbe-Buglé women from rural Panama. The bottom and top of each box represent the 25 and 75th percentiles, respectively; the horizontal line inside the box represents the median. Whiskers show the minimum and maximum values and dots represent outside values. Different lower case letters denote significant differences at *P* ≤ 0.05 (a significantly higher than b).

*T*-test comparisons of SFH Z-scores based on GA-specific cut-offs for eMAP, MAP < 70 mmHg, SBP/DBP < 100/60, and PP < 42 mmHg showed no significant differences. In order to determine whether lowest and highest centiles of the blood pressure measurements (SBP, DBP, MAP, PP) were associated with SFH, each BP measurement was included as an independent factor variable in separate models for SFH Z scores. Only PP emerged as significantly associated with SFH Z-score (Table 5). Compared to women whose PP was between the 10 and 90th centile, SFH-Z score was lower in women in the <10th centile but similar to those in the >90th centile (Figure 5).

**Table 5:** Multiple linear regression models of symphysis fundal height (SFH) Z-scores and pulse pressure (PP) in 177 pregnant Ngäbe-Buglé women from rural Panama with gestational age ≥ 16 wks.

**Adjusted Model for SFH-Z score^a^^,^^b^^,^^c^**	**Coefficient ± SE**	***P***	**95% CI**	**β**	**Overall Model**
PP <10th centile (<30 mmHg)	−2.85 ± 0.77	<0.0001	−4.37, −1.32	−0.262	*P* < 0.0001 Adj. *R*^2^ = 0.117
PP > 90th centile (>51.6 mmHg)	−0.06 ± 0.53	0.899	−1.12, 0.98	−0.009	
Weight for height classification	0.57 ± 0.26	0.029	0.06, 1.08	0.158	
Wood smoke (h/d)	−0.30 ± 0.09	0.002	−0.50, −0.11	−0.222	
Constant	−2.00 ± 0.60	0.001	−3.19, −0.80		

a *A three-level factor was used for our independent variable, pulse pressure (<10th centile, ≥ 10– ≤ 90th centiles, and > 90th centile), and comparisons were made against the base category (≥10– ≤ 90th centile). Adjusted for BMI category and wood smoke exposure (h/d)*.

b *n = 177. Missing data for SFH (15). Little's Chi-squared test for randomness of missing data, P = 0.466*.

*VIF = 1.02. Condition number = 15.45*.

c *SFH, symphysis-fundal height; PP, pulse pressure*.

## Discussion

The Ngäbe-Buglé indigenous community has one of the highest rates of adverse pregnancy outcomes in Panama, due in part to its remoteness and difficult access to health care, making it imperative from a public health perspective, to be better able to detect women at high risk. Compared with traditional reliance on SBP and DBP, we have observed that MAP and PP provided important information about the risk of both high and low blood pressure in our study population. High MAP has been validated as a risk factor for HDPs (58), and in fact, eMAP was the only BP measurement that allowed us to identify risk of HDPs in our MINDI cohort (11.3%). Furthermore, few studies have investigated the prevalence of low BP in marginalized communities. We identified a high prevalence of low BP, and importantly, low PP was associated with the lowest SFH for gestational age. These findings emphasize importance of routine blood pressure measurements in remote areas to identify women at risk of poor pregnancy outcomes. Consistent with our hypothesis, we also provide evidence that individual infections, nutrient deficiencies and cytokines, our markers of inflammation, differentially modified each blood pressure measurement, and may account for the normal values for SPB and DPB, despite evidence of high and low blood pressure based on MAP and PP. With regard to the presence of multiple infections, those that were associated with an anti-inflammatory (Th2) response (*Ascaris, Trichomonas*, scabies) were associated with lower blood pressure (SBP, DBP, MAP, and SBP/DBP < 100/60 mmHg) whereas those associated with a pro-inflammatory (Th1) response (hookworm, *Trichuris*, UTI) were associated with higher blood pressure measurements (SBP, DBP, and MAP). With regard to inflammation, we found that TNF-α, which is a hallmark cytokine for HDPs, was associated not only with higher SBP but also with higher MAP, supporting the use of MAP for early detection of women at risk of HDPs. In contrast, IL-17, known to increase with placental hypoperfusion, was negatively associated with PP. With regards to specific nutritional deficiencies, low protein and folic acid deficiency were associated with higher MAP and eMAP, respectively. Thus against the backdrop of low blood pressure in nearly 25% of the population, we report for the first time that higher intakes of MNS and animal source foods were associated with reduced likelihood of low blood pressure.

### Nutrition and Blood Pressure

There is growing evidence that blood pressure is modulated by several nutrients and not just sodium (59). Recently the INTERMAP Study (60) identified protein, insoluble fiber, phosphorus, calcium, magnesium, and non-heme iron as having inverse relationships with SBP and DBP. In our study, pregnant mothers consumed a poor diet leading to protein and micronutrient deficiencies, but were provided with a dietary supplement by the Ministry of Health (61) containing energy (400 kcal), protein (12.0 g), lipids (12–14 g), and several micronutrients (vitamins A, E, B_1_, B_2_, B_3_, B_6_, B_12_, folic acid, calcium, phosphorus, iron, zinc, and copper). Noteworthy among our findings was the observation that even though women in our study consumed less of the MNS than recommended, its intake was associated with a slightly higher SBP, DBP, MAP, and PP, which aligns with a previous MNS study showing that folic acid supplementation was also positively associated with SBP, DBP, MAP, and PP in a high-income setting (62). In our study, the MNS was also associated with decreasing the odds of BP < 100/60, suggesting that adequate intake of one or more of the nutrients included in the MNS might help normalize the low blood pressure in our population of pregnant women.

One characteristic of our population is the intake of diluted coffee. It is known that caffeine at dosages from by 80 to 300 mg can increase SBP by about 3–8 mmHg and DBP by about 4–6 mmHg (63). In our population, coffee concentrations had a median of 2.6 (range 6.5–24.5) mg/100 mL, and coffee intake was associated not with higher but with lower MAP, and only in the first trimester. A beneficial effect of polyphenols contained in coffee (64, 65) or a higher intake of fluids with positive effect on cardiovascular health (66) may account for this association.

Several studies have examined the consequences of maternal dietary protein intake on pregnancy outcomes, particularly SGA. Although a Cochrane review concluded that there is no justification for prescribing high-protein nutritional supplements to pregnant women (67), a more recent meta-analysis of studies from low-medium income countries found that balanced protein-energy supplementation of undernourished women significantly improved birthweight (68). Although the link between maternal protein malnutrition and hypertension in the offspring has been established (69), one study with Dutch women showed that protein intake-related acid load was not associated with HDPs, in protein-replete women (70). However, a higher vegetable protein/potassium ratio was associated with lower DBP (70), which is consistent with a meta-analysis in non-pregnant populations from developed countries that showed that increased intake of dietary protein relative to carbohydrate was associated with lower blood pressure (71). In our marginalized community, there was clinical evidence of dietary protein deficiency with 26% of mothers having low RBP and 85% having low B_12_, but our finding that higher intakes of animal source foods reduced the odds of low blood pressure agrees with observations from developing settings where higher protein intakes in the context of carbohydrate-rich diets could decrease blood pressure (71, 72). Our study is the first to observe an association of higher protein intake with the reduced odds of hypotension in marginalized communities.

Another nutrient deficiency that emerged as increasing the odds for elevated MAP was folic acid. We had previously shown that folic acid deficiency was positively associated with elevated CRP in lactating women in our population (32), which aligns with evidence that folic acid deficiency may promote increased BP probably as consequence of inflammation (73). Folic acid supplementation for the prevention of neural tube defects and low birthweight has been studied (74), but its use for the prevention of HDPs is still controversial. Although folic acid supplementation did not change SBP or DBP in women from Iran (75) or The Netherlands (62), and although folic acid levels were similar between normal and hypertensive pregnancies in India (76), it has been reported that folate supplementation decreased the risk of HDPs in populations from China (77) and Korea (78). The association of folic acid deficiency with increased odds of eMAP points toward a benefit of folic acid supplementation for the prevention of HDPs and aligns with the significant positive correlations of serum homocysteine with high MAP in women with pregnancy complicated by HDP in Pakistan (17).

### BP and Infections

A particularly intriguing and novel observation was the bi-directional nature of associations between infections and blood pressure indicators. Hookworm was associated with higher SBP, DBP, and MAP and also was associated with lower odds of low blood pressure, and *Trichuris* was associated with increased odds of eMAP. The only data linking this type of infection with blood pressure comes from studies with an intestinal nematode *Strongyloides venezuelensis* that is used as a hookworm model in rodents. This nematode infection was found to increase both SBP and MAP in rats, possibly reflecting the impact of nematode-induced inflammation on blood pressure (79). These results are consistent with the higher blood pressure observed in our pregnant women who were infected with hookworm. In contrast, a third intestinal nematode, *Ascaris*, as well as the scabies mite and the vaginal protozoan, *Trichomonas*, were associated with lower blood pressure and *Ascaris* also increased the odds of low blood pressure.

The contrasting direction of association for *Ascaris*, scabies and trichomoniasis with blood pressure compared with hookworm and *Trichuris* is consistent with the distinctiveness of the inflammatory and immunological responses that they induce. First, in these same pregnant women, CRP was shown to be negatively associated with *Ascaris* but positively associated with hookworm (32), suggesting that *Ascaris* exerted an anti-inflammatory influence whereas hookworm induced a pro-inflammatory response. Second, intestinal nematodes including *Ascaris* (80), scabies mites (81), and *Trichomonas vaginalis* (82, 83) are able to modulate the host immune response with production of classical Th2 cytokines, IL-4, IL-5, and IL-13, mastocytosis, eosinophilia, IgE, and alternatively-activated macrophages known to play a critical role in tissue repair (84). In contrast, hookworm releases molecules that down-regulate the strong Th2 response through a mixed Th2/Th1 response with elevation of pro-inflammatory cytokines including TNF-α (85–87) similar to the pro-inflammatory response observed in response to chronic low dose infection of mice with *Trichuris muris* (88). Taken together, it is not surprising that the association with blood pressure differed for *Ascaris*, scabies, and trichomoniasis compared with hookworm and *Trichuris*, and it is likely that this was linked to the associated anti-inflammatory and pro-inflammatory responses, respectively.

### Blood Pressure, Cytokines, and Inflammation

TNF-α was positively associated with SBP, MAP, and PP. Concentrations of TNF-α are known to increase under placental hypoperfusion, and TNF-α is able to increase BP by activating humoral and endothelial factors (89). As TNF-α has been consistently found to be elevated in women with HDPs (90–93), as our TNF-α values were within ranges associated with HDPs, and as we observed a positive association between TNF-α and MAP, our data suggest that eMAP may be a useful early indicator of women at risk for HDPs in MINDI populations.

IL-17 has also been linked to hypertension not only by producing pro-inflammatory and endothelial damage, but also by up-regulating transport channels in the tubules of the kidney (90). This raises an interesting question regarding hemodynamics in these pregnant women, given that hypoproteinemia was common. It is known that in protein malnutrition, there is an increase in renal vascular resistance, renal blood flow and sodium excretion (94). Therefore, the pro-inflammatory response measured through IL-17 might be helping to compensate for hypovolemia by increasing sodium reabsorption and BP, at the expense of perfusion indicated by PP (3). In support of this hypothesis, recent studies have shown that the number of Th17 cells or the concentration of IL-17 is higher in women with hypertension during pregnancy (95, 96), in agreement with our observation that IL-17 was associated with decreased odds of hypotension. Also, experimental studies have shown that Th17 cells from animals with reduced uterine perfusion are able to increase BP and to induce IUGR when injected to normal pregnant animals (97), in agreement with our association of higher IL-17 with decreased PP, and our further observation that low PP was associated with small SFH.

### BP Measurements and Symphysis-Fundal Height

Although ultrasound is considered the gold standard for detecting SGA (27), it was not available for our population at the time of the study, and clinicians in the field used SFH as equivalent for estimating fetal growth, based on Pan American Health Organization standards that have reported a sensitivity of 52% and specificity of 92% for the detection of SGA (28). Despite the large sample size of the INTERGROWTH study, authors did not consider it appropriate to make recommendations about the cutoff values for SGA (29). We overcame this limitation by using the continuous variable of SFH Z scores as our outcome in order to describe its associations with BP measurements.

Hypertension during pregnancy is a known cause of IUGR (98), and although we found women at risk of HDPs based on elevated MAP, none of the blood pressure indicators was associated with low SFH. This differs from several other studies. Higher PP was associated with lower birthweight in a cohort of 50 normotensive women from the Netherlands (99). Also, European (100) and Asian studies (101) have shown that elevated SBP and DBP were associated with impaired fetal growth. Although few studies have addressed hypotension as a potential risk factor for SGA, we found that low PP (but not low SBP, DBP, or MAP) was associated with SGA fetuses in our cohort of women participating in normal pregnancy follow up. It is known that women who do not achieve the physiologic increase of blood volume are prone to HDPs, fetal growth restriction and SGA (102, 103). The risk of stillbirth has been reported to be higher in women with low DBP and low MAP (9), and low BP defined as DBP < 80 mmHg was associated with preterm birth and SGA although this association was lost after controlling for maternal age, BMI, socio-economic status, and race (8). Our findings demonstrate that hypotension may be an unrecognized factor contributing to impaired SFH in our MINDI cohort.

### Limitations

Despite the increased need for research to understand major health issues in remote settings, a recent review shows that among clinical research studies, 3.27% are dedicated to women, 1.72% to the poor, 1.66% to rural residents and 1.55% to visible minorities (104). Our study may not be comparable to populations where most clinical research originates, but it reflects the reality of an important proportion of the world population, where women have limited access to technology and where the utility of simple biomarkers such as blood pressure and SFH needs to be maximized.

Data on birth outcomes was not available given that most women delivered at home, a limitation that is shared by clinicians in their daily practice during routine pregnancy follow-ups. Just as field clinicians, we needed to make the best use of available resources to identify pregnant women at risk. Despite this limitation, our comprehensive database allowed us to identify novel associations of BP measurements with MINDI and to provide evidence that MAP and PP, together with SFH, may help in the identification of women who are at high risk to develop complications.

## Conclusion

Our results demonstrate that routine blood pressure measurements, in particular eMAP, which reflects overall exposure to multiple infections, nutritional deficiencies and inflammation, can be used to assess at-risk pregnancies in remote settings where SBP and DBP were unable to detect risk of HDPs. Our findings also showed that protein deficiency was associated with higher MAP and that folate deficiency was associated with increased odds of eMAP, suggesting that current folic acid and protein supplementation need to be strengthened to meet the needs during pregnancy. Our novel finding of associations of infections with SBP, DBP, and MAP call for the need to consider that *Ascaris, Trichomonas* and scabies may lower BP, whereas hookworm, *Trichuris* and UTI may increase BP, and have implications for deworming programs during pregnancy. Furthermore, given that neither SBP, DBP, nor MAP were associated with SFH, but PP was, the association of low PP with smaller SFH makes it a promising indicator for women at risk of adverse pregnancy outcomes in remote areas where sonography is not available.

## Data Availability Statement

The datasets for this article are not publicly available because participants did not sign informed consent that data will be publicly available, neither was this possibility discussed with Ethical Boards in Panama or Canada. Requests to access the datasets should be directed to Dr. Kristine G. Koski (kristine.koski@mcgill.ca).

## Ethics Statement

The studies involving human participants were reviewed and approved by Gorgas Memorial Institute Research Bioethics Committee (Panama) and McGill University Research Ethics Board. Written informed consent to participate in this study was provided by the participants' legal guardian/next of kin.

## Author Contributions

DG-F contributed to study design and conceptual framework, data collection and statistical analysis, and writing of the final manuscript. EP, DR, OS, and EM contributed to the study design, coordinated field and laboratory analyses, read, and approved the final manuscript. MS contributed to the study design, conceptual framework, and read and approved the final manuscript. KK contributed to study design and conceptual framework, data collection, and statistical analysis and writing of the final manuscript. EM, MS and KK were involved in funding the study.

### Conflict of Interest

The authors declare that the research was conducted in the absence of any commercial or financial relationships that could be construed as a potential conflict of interest.

## Abbreviations

BP: blood pressure
CRP: C-reactive protein
DBP: diastolic blood pressure
eMAP: elevated mean arterial pressure
GA: gestational age
HDPs: hypertensive disorders of pregnancy
IFN: Interferon
IL: interleukin
IUGR: intra-uterine growth retardation
MAP: mean arterial pressure
MNS: multiple nutrient supplement
NLR: neutrophil/lymphocyte ratio
PP: pulse pressure
RBC: red blood cells
RBP: retinol-binding protein
SBP: systolic blood pressure
SFH: symphysis-fundal height
SGA: small-for-gestational-age
TNF: tumor necrosis factor
USG: urinary specific gravity
WBC: white blood cells.
