# Supplementary material for: Identification of High-Risk Pregnancies in a Remote Setting Using Ambulatory Blood Pressure: The MINDI Cohort
Source: Front Public Health. 2020 Mar 24;8:86. doi: 10.3389/fpubh.2020.00086 (PMC7121149; doi:10.3389/fpubh.2020.00086)
Supplement: Supplementary file 1 [file Data_Sheet_1.pdf]

Supplementary Material

Supplementary Tables

Table S1 Multiple linear regression models for systolic (SBP), diastolic (DBP) blood pressure and mean arterial pressure (MAP) in the dataset of indigenous pregnant Ngäbe-Buglé women (n= 206-207) without intestinal nematode data.

**SBP**[1]	**Coef. ± SE**	**P**	**95% CI**	**β**	**Overall Model**
Gestational age, wk	-0.006 ± 0.07	0.927	-0.14, 0.13	-0.006	P< 0.0001<br>Adj. R^2= 0.170
Weight for height category	3.28 ± 1.26	0.010	0.78, 5.77	0.176	
Multiple nutrient supplement, tbsp./d	1.62 ± 0.45	<0.0001	0.72, 2.52	0.248	
IL17	-0.20 ± 0.08	0.024	-0.37, -0.02	-0.203	
TNFα	0.32 ± 0.11	0.004	0.10, 0.53	0.263	
Hematocrit quantile[4]	1.28 ± 0.60	0.035	0.09, 2.46	0.139	
Scabies, presence	-3.86 ± 1.75	0.028	-7.32, -0.41	-0.143	
Urinary bacteria (+)	-1.94 ± 1.14	0.092	-4.20, 0.32	-0.109	
Trichomonas, presence	-3.81 ± 1.57	0.016	-6.92, -0.70	-0.159	
Constant	95.79 ± 3.81	<0.0001	88.27, 103.3		
**DBP**[2]	**Coef. ± SE**	**P**	**95% CI**	**β**	**Overall Model**
Gestational age, wk	0.05 ± 0.06	0.416	-0.07, 0.16	0.057	P= 0.0001<br>Adj. R^2= 0.113
Urinary gravity >1020	3.32 ± 1.25	0.009	0.85, 5.80	0.175	
Weight for height category	2.30 ± 1.05	0.029	0.23, 4.37	0.150	
Multiple nutrient supplement, tbsp./d	0.82 ± 0.38	0.034	0.06, 1.58	0.152	
Hematocrit, quantile[4]	1.30 ± 0.50	0.010	0.31, 2.28	0.172	
Trichomonas, presence	-2.22 ±1.34	0.100	-4.86, 0.42	-0.112	
Vitamin D< 50 nmol/L	-2.03 ± 1.16	0.082	-4.32, 0.26	-0.115	
Constant	53.07 ± 3.09	<0.0001	46.97,59.17		
**MAP**[3]	**Coef. ± SE**	**P**	**95% CI**	**β**	**Overall Model**
Gestational age, wk	0.004 ± 0.05	0.939	-0.11, 0.12	0.005	P< 0.0001<br>Adj. R^2= 0.168
Weight for height category	2.87 ± 0.99	0.004	0.92, 4.83	0.194	
Urinary gravity >1020	3.05 ± 1.18	0.011	0.71, 5.39	0.167	
Multiple nutrient supplement, tbsp./d	1.03 ± 0.36	0.005	0.31, 1.74	0.198	
TNFα	0.10 ± 0.06	0.114	-0.02, 0.22	0.103	
Hematocrit, quantile[4]	1.34 ± 0.48	0.006	0.39, 2.29	0.185	
Trichomonas, presence	-2.26 ± 1.25	0.072	-4.74, 0.20	-0.119	
Folic acid <10 nmol/L	3.82 ± 1.27	0.003	1.31, 6.32	0.200	
Retinol-binding protein <30 mg/L	1.83 ± 1.22	0.136	-0.58, 4.25	0.099	
Constant	63.0 ± 2.9	<0.0001	57.3, 68.7		

[1] n= 206, Variance inflation factor= 1.29. Condition number= 17.73. Variables that were included but had a P>0.10: IL13 (pg/mL), presence of caries, wood smoke exposure.
[2] n= 207. Variance inflation factor= 1.08.Condition number= 15.72. Variables that were included but had a P>0.10: presence of *Trichomonas*, IL13 (pg/mL), taking iron supplements (y/n), hemoglobin (g/L).
[3] n= 206. Variance inflation factor= 1.10. Condition number= 16.35. Variables that were included but had a P>0.10: TNFα (pg/mL), retinol-binding protein <30 mg/L, vaginal yeast (+), vitamin D<50 nmol/L, urinary protein (+), hemoglobin (g/L), IL13 (pg/mL)
[4] Hematocrit was divided into quantiles according to its distribution in our population: <25th quantile, 25th – 50th, 50th – 75th and >75th quantiles (<33.2%, 33.2-35%, 35-36.9% and >36.9%, respectively)

Table S2 Multiple logistic regression models for elevated MAP (eMAP) and low blood pressure (SBP<100 and DBP <60 mmHg) in the dataset of indigenous pregnant Ngäbe-Buglé women (n= 212), where data on intestinal nematodes were not included.

**eMAP[1]**	**OR ± SE**	**P**	**95% CI**	**Overall Model**
Age	1.10 ± 0.04	0.004	1.03, 1.18	
Folic acid <10 nmol/L	3.48 ± 1.84	0.018	1.23, 9.80	
Multiple nutrient supplement, tbsp./d	1.28 ± 0.18	0.078	0.97, 1.68	P= 0.0001
Neutrophil/Lymphocyte ratio	0.62 ± 0.18	0.099	0.35, 1.09	Pseudo R^2= 0.198
IL1β, pg/mL	1.11 ± 0.05	0.014	1.02, 1.21	
IL17, pg/mL	0.93 ± 0.04	0.084	0.86, 1.01	
Constant	0.01 ± 0.02	0.001	0.001, 0.17	
**Low blood pressure[2]**	**OR ± SE**	**P**	**95% CI**	**Overall Model**
Gestational age, wk	1.00 ± 0.02	0.935	0.95, 1.05	
Vitamin D, nmol/L	0.97 ± 0.01	0.049	0.95, 0.99	
Field work, h/d	0.83 ± 0.06	0.009	0.72, 0.95	
Iron supplementation, months	0.80 ± 0.09	0.061	0.64, 1.00	
Multiple nutrient supplement, tbsp./d	0.62 ± 0.10	0.005	0.44, 0.87	P< 0.0001
Folic acid <10 nmol/L	0.31 ± 0.15	0.019	0.12, 0.83	Pseudo R^2= 0.200
Scabies, presence	2.23 1.02	0.079	0.91, 5.45	
Hematocrit, quantile[3]	0.64 ± 0.11	0.010	0.46, 0.90	
IL17, pg/mL	0.91 ± 0.03	0.005	0.85, 0.97	
INFγ, pg/mL	1.06 ± 0.03	0.026	1.01, 1.11	
Constant	13.48 ± 13.3	0.009	1.94, 93.71	

[1] eMAP defined as >87 mmHg between weeks 10-18, >84 mmHg in weeks 18-34, and >86 mmHg after week 34 (Reference 41).
n= 207. Variance inflation factor= 1.16. Condition number= 11.34. Variables that were included but had a P>0.10: urinary bacteria ≥2+, presence of vaginal yeast, retinol-binding protein <30 mg/L, mean corpuscular volume (fL), taking iron supplements (no=0, yes=1), weight for height classification.
[2] Low blood pressure defined as SBP <100 and DBP <60 mmHg (Reference 2).
n=212. Variance inflation factor= 1.39. Condition number=15.76. Variables that were included but had a P>0.10: green/leafy vegetables (portions/wk), animal source foods (portions/wk), body mass index (kg/m^2).
[3] Hematocrit was divided into quantiles according to its distribution in our population: <25th quantile, 25th – 50th, 50th – 75th and >75th quantiles (<33.2%, 33.2-35%, 35-36.9% and >36.9%, respectively)

Table S3 Stepwise multiple linear regression models for mean arterial pressure (MAP, mmHg) by trimester. Models were ran with and without intestinal nematode data.

**MAP in the 1st trimester[1]**	**Coef. ± SE**	**P**	**95% CI**	**β**	**Overall Model**
Coffee, cups/d	-2.24 ± 1.03	0.040	-4.38, -0.11	-0.316	P=0.0002<br>Adjusted R^2= 0.47
Red blood cell count, centiles[6]	6.67 ± 1.52	<0.0001	3.52, 9.82	0.637	
Constant	64.5 ± 4.12	<0.0001	56.0, 73.1		
**MAP in the 2nd trimester[2]**	**Coef. ± SE**	**P**	**95% CI**	**β**	**Overall Model**
Multiple nutrient supplement, tbsp/d	1.93 ± 0.72	0.009	0.50, 3.37	0.266	P<0.0001<br>Adjusted R^2= 0.304
Folic acid, nmol/L	-0.23 ± 0.10	0.022	-0.42, -0.03	-0.223	
Hematocrit, quantiles[5]	2.38 ± 0.77	0.003	0.85, 3.90	0.300	
Urinary gravity ≥1020	5.10 ± 1.89	0.009	1.33, 8.87	0.263	
Urinary bacteria, +/high power field	-3.80 ± 1.50	0.013	-6.78, -0.81	-0.257	
Constant	73.64 ± 3.07	<0.0001	67.53, 79.75		
**MAP in the 3rd trimester[3]**	**Coef. ± SE**	**P**	**95% CI**	**β**	**Overall Model**
Folic acid <10 nmol/L	2.85 ± 1.60	0.077	-0.32, 6.02	0.167	P=0.0015<br>Adjusted R^2= 0.119
Weight for height classification	4.37 ± 1.37	0.002	1.65, 7.09	0.329	
Multiple nutrient supplement, tbsp/d	1.35 ± 0.43	0.003	0.48, 2.21	0.318	
Constant	63.58 ± 3.45	<0.0001	56.74,70.43		
**MAP in the 3rd trimester, including nematodes[4]**	**Coef. ± SE**	**P**	**95% CI**	**β**	**Overall Model**
Age, centiles	2.94 ± 1.38	0.037	0.18, 5.71	0.244	P=0.0005<br>Adjusted R^2= 0.247
Weight for height classification	3.49 ± 1.60	0.034	0.28, 6.70	0.288	
Multiple nutrient supplement, tbsp/d	1.15 ± 0.49	0.022	0.17, 2.13	0.305	
Ascaris, presence	-5.11 ± 1.78	0.006	-8.78, -1.55	-0.325	
Constant	62.07 ± 4.57	<0.0001	52.91, 71.23		

[1] n= 26. Variance inflation factor= 1.00. Condition number= 7.38. Variables that were included but had a P> 0.05: Basophil centiles, Multiple nutrient supplement (tbsp/d), weekly portions of green-leafy vegetables.
Intestinal nematodes did not enter preliminary models for MAP in the first trimester.

[2] n= 79. Variance inflation factor= 1.04. Condition number= 8.90. Variables that were included but had a P> 0.05: IL6 (pg/mL), platelets, IL4 (pg/mL), IL13 (pg/mL), elevated CRP, presence of *Trichomonas*.
Ascaris entered a preliminary model, but did not enter the final model (not shown).

[3] n= 102. Variance inflation factor= 1.04. Condition number= 9.79. Variables that were included but had a P> 0.05: TNFα (centiles)[6], age (yr), vitamin D<50 nmol/L, IL1β (pg/mL), INFγ (pg/mL), urinary gravity <1020

[4] n= 61. Variance inflation factor= 1.07. Condition number= 11.91. Variables that were included but had a P> 0.05: INFγ (pg/mL), hookworm (presence), urinary gravity ≥1020, folic acid <10 nmol/L, vitamin D< 50 nmol/L, IL1β (centile)[6], TNFα (centile)[6].

[5]Hematocrit was divided into quantiles according to its distribution in our population: <25th quantile, 25th – 50th, 50th – 75th and >75th quantiles (<33.2%, 33.2-35%, 35-36.9% and >36.9%, respectively).
[6] Transformation of continuous variables into centiles (<25th, 25th-75th and >75th centiles) was performed when the continuous variable showed a non-linear association.
